# Human rights and humanitarian forensic action: the experience in Uruguay

**DOI:** 10.1080/20961790.2022.2052591

**Published:** 2022-05-06

**Authors:** Hugo Rodríguez Almada, Frances Borches Duhalde, Victoria Iglesias Salaverría

**Affiliations:** Department of Legal Medicine and Forensic Sciences, Faculty of Medicine, Universidad de la República, Uruguay

## Introduction

The daily practice of Forensic Medicine and Forensic Sciences is closely related to human rights, as it implies the study of phenomena such as violent deaths, sexual crimes, gender-based violence, different forms of child abuse, torture, deaths in custody and patients’ rights. The very ethics of the professional practice, as agreed in the Ethical Principles Guidelines of the Red Iberoamericana de Instituciones de Medicina Legal y Ciencias Forenses (Ibero-American Network of Institutions of Legal Medicine and Forensic Sciences), involves the core axiological foundation of respect for human rights, including those of all persons with whom professionals interact in their work, whether primary victims, secondary victims or perpetrators [1].

Humanitarian Forensic Action (HFA) is considered a new field of Forensic Medicine and Forensic Sciences [2].

According to the International Court of Justice, humanitarian action is defined as the activities carried out by organisations and individuals “to prevent and alleviate human suffering wherever it may be found” and “to protect life and health and to ensure respect for the human being” (alive or dead) [3].

One might ask, then, what does HFA propose that is new? According to Tidball-Binz [3], HFA is “the application of forensic science to humanitarian activities”. According to the International Committee of the Red Cross (ICRC), these activities “seek to alleviate human suffering and protect the dignity of all victims of armed conflict and catastrophes, carried out in a neutral, impartial and independent manner, free of charge and framed under International Humanitarian Law” [2]. The recovery and identification of the remains of victims of extrajudicial executions in the Spanish Civil War or of Argentine soldiers in the Islas Malvinas/Falkland Islands conflict, and the management of dead bodies in the context of major humanitarian disasters, such as the 2010 earthquake in Haiti, the Ebola epidemic in Africa, or the humanitarian crisis caused by the drowning in the Mediterranean Sea of hundreds of people trying to emigrate from Africa to Europe, are some examples that clearly illustrate the need for the development of this field of forensic action [2–6].

The definition of HFA warrants the following comments:

- In addition to the forensic management of post-armed conflict settings and the consequences of natural disasters, HFA encompasses other issues, including monitoring detention centres, diagnosis and documentation of torture and investigation of deaths in custody [2]. International documents such as the Istanbul Protocol and the Minnesota Protocol have been instruments of good practice for HFA, even before the theoretical foundations of this field of action were established [2,7,8].
- It has been asserted that HFA, in contrast to forensic work in the judicial context, does not seek to determine legal responsibilities but rather involves humanitarian action that aims to alleviate suffering and protect dignity. Although not primarily aimed at criminal prosecution, HFA may also entail a legal dimension. The work of the Argentine Forensic Anthropology Team—considered the first organised and documented HFA experience [3]—shows the difficulty of establishing a clear-cut distinction between the judicial and humanitarian aspects. Conversely, forensic practice carried out in the context of a criminal investigation usually involves a humanitarian action for the benefit of victims, families and the community. For example, by determining the cause, manner and circumstances of the death of a victim of torture that occurred 40 or 50 years ago, one is contributing to the fulfilment of human rights standards—such as symbolic reparation—that alleviate suffering and restore dignity.
- The *neutral* nature of HFA conveyed in its definition should be clarified to avoid misinterpretations. It reveals the imprint of the ICRC, which has played a decisive role in its theoretical development and, above all, in its practice in the field [3]. The neutral stance relates to an operational need in contexts of armed conflict and to facilitate the involvement of governments in humanitarian tasks. It does not, however, suggest keeping an equidistant position between victims and perpetrators or being indifferent to crimes against humanity or any form of human rights violation [1,9,10].
- The nonprofit nature of HFA merits discussion. On the one hand, HFA is not a task for volunteers; instead, it requires the participation of qualified and trained professionals. On the other hand, turning a good cause into a business must be avoided to preserve HFA’s original and authentic purpose; it would be unacceptable for experts to benefit from humanitarian emergencies.

## HFA in Uruguay

Forensic Medicine and Forensic Sciences acknowledge universal principles, both from the technical and ethical perspectives. At the same time, the development and organisational methods of these principles differ, depending on each country’s singularities [11]. The same applies to Forensic Medicine and Forensic Sciences regarding humanitarian action.

Uruguayan Forensic Medicine evolved in a particular historical, geographical, social, political, legal and institutional context, different from that of countries in other continents and even in the same region. Because Uruguay is not accustomed to major catastrophes, its response services are not as well developed as in other countries. Forensic disciplines have also followed a very uneven evolution, marked by this peculiarity. Determining factors of the poor progress of forensic work in Uruguay [11] include the lack of a critical mass of educated and trained professionals in some forensic disciplines and a sustained shortage of financial resources.

Although the country has not experienced humanitarian emergencies caused by major mass disasters, Uruguayans have had to deal with persistent humanitarian crises, such as those caused by State terrorism (1973–1985), and endemic crises, such as the ongoing violation of the rights of persons incarcerated in the prison system.

In Uruguay, Forensic Medicine and Forensic Sciences have their pioneering institution in the Faculty of Medicine, Universidad de la República. The Chair of Forensic Medicine—today called the Department of Legal Medicine and Forensic Sciences—was founded in 1877 and, since then, has led the development of the discipline in the country [11].

Its institutional mission is *“Implementing quality teaching of Forensic Medicine and Forensic Sciences at undergraduate and graduate levels. Promoting research as a contribution to the improvement of the health and justice system. Developing assistance, counseling, outreach programmes and activities, with an emphasis on academic quality, forensic ethics, and the promotion of human rights”.*

This practice is part of an institutional context that is also unique. The legal regulations governing the Universidad de la República provide that it is a state institution, but that—by constitutional mandate—it is autonomous from the executive branch. Among its purposes, Law No. 12549, enacted in 1958, establishes “the defense of moral values and the principles of justice, freedom, social welfare, the rights of human beings and the democratic republican form of government”.

In this particular context, a “Uruguayan model” of HFA has been developed. It is based on international documents, including the Istanbul Protocol, the Minnesota Protocol and the ethical principles guidelines of the Red Iberoamericana de Instituciones de Medicina Legal y Ciencias Forenses. We have also developed some methodologies appropriate to the particular features of the country, which might also be suitable for other national contexts.

## Main lines of work

The Department of Legal Medicine and Forensic Sciences has devised and maintained several lines of action in response to demands such as those mentioned above. In some cases, this work has been formalised through agreements with public institutions, such as those with the National Human Rights Institution and Ombudsman (Institución Nacional de Derechos Humanos y Defensoría del Pueblo, INDDHH), which includes the National Mechanism for the Prevention of Torture (MNP), and with the Ministry of Education and Culture to comply with Law No. 18596 (reparation for victims of State illegitimate actions). In other cases, the Department acts at the request of victims, families, prosecutors and courts. At all times, humanitarian or human rights forensic work contributes to the education and training of young forensic professionals.

### National mechanism for the prevention of torture

In 2008, Law No. 18446 created the INDDHH as an institution within the Legislative Branch, independent and separate from the Executive and the Judiciary, responsible for the protection of human rights in every aspect to every extent possible within the constitutional framework.

From its inception, the INDDHH required the collaboration of the Department of Legal Medicine and Forensic Sciences. Such collaborations were initially performed without charge; in 2016, an agreement formalized funding the provision of technical advice, to the MNP in particular.

As part of this agreement, experienced professionals, together with young people undergoing training, have assisted MNP specialists in monitoring institutions where people are deprived of liberty in a broad sense (e.g. police facilities, penitentiary units, detention centres for teenagers in conflict with criminal law, psychiatric institutions and protection centres for children and teenagers).

During the inspection of these places, the medicolegal perspective acquires great relevance for assessing how persons are and remain in the context of deprivation of liberty, as well as for the investigation and documentation of potential situations of torture and other cruel, inhuman or degrading treatment or punishment, or other human rights violations. This task of assistance includes medicolegal reports that contribute to the preparation of recommendations seeking to strengthen the protection of individuals and to minimize the risks of human rights violations.

These actions have three fundamental characteristics:

- They are carried out in an absolutely independent manner, including from the INDDHH and the MNP.
- Participants do not receive any additional compensation for this service, which is part of their university work obligations. Compensations granted by the INDDHH are passed on to the Department of Legal Medicine and Forensic Sciences.
- All these activities constitute learning opportunities for professionals in training.

### The prison system and deaths in custody

Law No. 17684 (2003) created the Parliamentary Commissioner for the Penitentiary System with the main function of advising the Legislative Branch regarding its duty to monitor compliance with legal regulations concerning persons deprived of their liberty by judicial decision. The Commissioner’s legal powers include promoting the rights of persons deprived of their liberty, carrying out the studies and preparing the reports deemed appropriate for the best performance of the duties of the office.

In conducting this work, which is of considerable humanitarian significance, the advice of different areas of the Universidad de la República is sought, including the Department of Legal Medicine and Forensic Sciences of the School of Medicine.

This collaboration includes the clinical assessment of persons deprived of their liberty when there are suspicions or allegations of maltreatment, negligence in the care of persons with physical or mental illnesses, or inappropriate incarceration in a common prison. In addition, the Department studies and systematises individual cases of deaths in custody and makes recommendations to the Office of the Parliamentary Commissioner for their prevention [12,13].

In September 2021, an agreement was signed between the Legislative Branch and the Universidad de la República to allow the financing of these activities, which have been carried out in recent years free of charge. As in the case of providing advice to the MNP, reports are prepared in an independent manner, and there is no additional compensation for such work, which is considered a learning opportunity for professionals undergoing training.

### Victims of State terrorism

Since 2007, the Department of Legal Medicine and Forensic Sciences has also responded to different requests in the study of numerous deaths in custody that took place during the period of State terrorism. Although the skeletal remains of victims were occasionally available to respond to the requests, in most instances, this was not the case. In response, the method known as *historical autopsy* was developed, defined as the forensic investigation of the cause, manner and circumstances of a death bearing historical, humanitarian or judicial interest when there is no direct access to the corpse or its skeletal remains. Conclusions are based on the study and critical, harmonic, hierarchical and objective interpretation of the information provided by documents and testimonies [14]. At first, this method was applied only in cases of purely historical or humanitarian interest at the request of relatives but has since been requested as expert evidence by prosecutors and/or courts [15].

In 2009, Law No. 18596 was enacted, whereby the responsibility of the State regarding the violation of fundamental rights of individuals was established and violations of human rights, from June 27, 1973 to February 28, 1985 in particular, were acknowledged. The law also established pecuniary, health and/or symbolic reparations for victims. To qualify for pecuniary reparations, the law requires that the victim has suffered “very serious injuries”. All these reports were commissioned to the Department of Legal Medicine and Forensic Sciences [16]. In the case where victims are survivors, humanitarian action must prioritise their non-revictimization in the process of seeking reparation.

Recently, the judicial system has requested dozens of reports on the adequacy of certain methods of torture practised during the period of State terrorism to inflict “serious injuries” or “very serious injuries” on the victims [17].

## Conclusion

As propounded by Ubelaker, “in many areas of the contemporary world, problems emerge related to humanitarian issues and possible violations of human rights” and “modern forensic science can contribute in many positive ways to the resolution of these issues” [18].

Although these problems are global, each region and country presents its own particular circumstances. How these challenges are faced also involves issues that are common to global Forensic Science, as well as issues concerning *ad hoc* institutional and organisational aspects. Forensic professionals in every country must study and learn from the progress and experiences of others to do a better and more efficient job without uncritically copying other models designed for different realities.

In Uruguay, HFA—understood as the work of forensic professionals to prevent and alleviate human suffering wherever it is found, to protect life and health and guarantee respect for the human being (alive or dead)—has developed along with and inseparably from its commitment to achieving the human rights standards of truth, justice, memory, reparation and a guarantee that violations will not happen again.

The development of this action has been nurtured by regional and international knowledge, experience and recommendations, critically adapted to the Uruguayan reality. It has also contributed to the development of forensics investigations methods, such as the historical autopsy, which has demonstrated its humanitarian and judicial value.

This “Uruguayan model” is nongovernmental, yet inserted within the public sector and closely related to academia, adopting an integral paradigm that involves teaching, scientific research and practical application in the environment to meet the social demands in this field. All activities are performed on a nonprofit basis and do not include any financial benefit for the professionals who carry them out.


Hugo Rodríguez Almada, Frances Borches Duhalde and Victoria Iglesias Salaverría 
Department of Legal Medicine and Forensic Sciences, Faculty of Medicine, Universidad de la República, Uruguay


